# Supplementary material for: Machine learning identifies molecular regulators and therapeutics for targeting SARS‐CoV2‐induced cytokine release
Source: Mol Syst Biol. 2021 Sep 6;17(9):e10426. doi: 10.15252/msb.202110426 (PMC8420181; doi:10.15252/msb.202110426)
Supplement: Supplementary file 1 — Expanded View Figures PDF [file MSB-17-e10426-s004.pdf]

Expanded View Figures

Figure EV1. SARS-Cov-2 Spike subunit S1 protein causes a significant increase in the expression of a panel of cytokines in myeloid cells.

A Plot showing dose-dependent increase in *IL-1b* expression in THP1-derived macrophages in response to S1 protein.
B S1 RBD domain does not activate THP1 cells. THP1 cells were stimulated with indicated protein (1 μg/ml) or PBS for 24 h. Gene expression was measured by qPCR.
C SARS-Cov-2 spike protein activates mouse macrophages *in vitro*. Plots showing changes in the expression of cytokines in mouse Raw264.7 macrophages upon treatment with different domains of S1 subunit at 1 μg/ml for 24 h. *Right*, a schematic showing domain structure of full-length S1 protein and RBD only.
D The NTD is more divergent than the RBD of SARS-Cov-2. Plot shows sequence homology of NTD and RBD region of the SARS-Cov-2 spike protein with other coronaviruses.
E Spike protein from endemic viruses does not activate THP1-derived macrophages. THP1 cells were stimulated with indicated protein (1 μg/ml) or PBS for 24 h. Cytokine release was measured by Luminex.

Data information: in (A-C, E), data are presented as means of 3 technical replicates. Error bars denote SEM.

 ©2021 The Authors

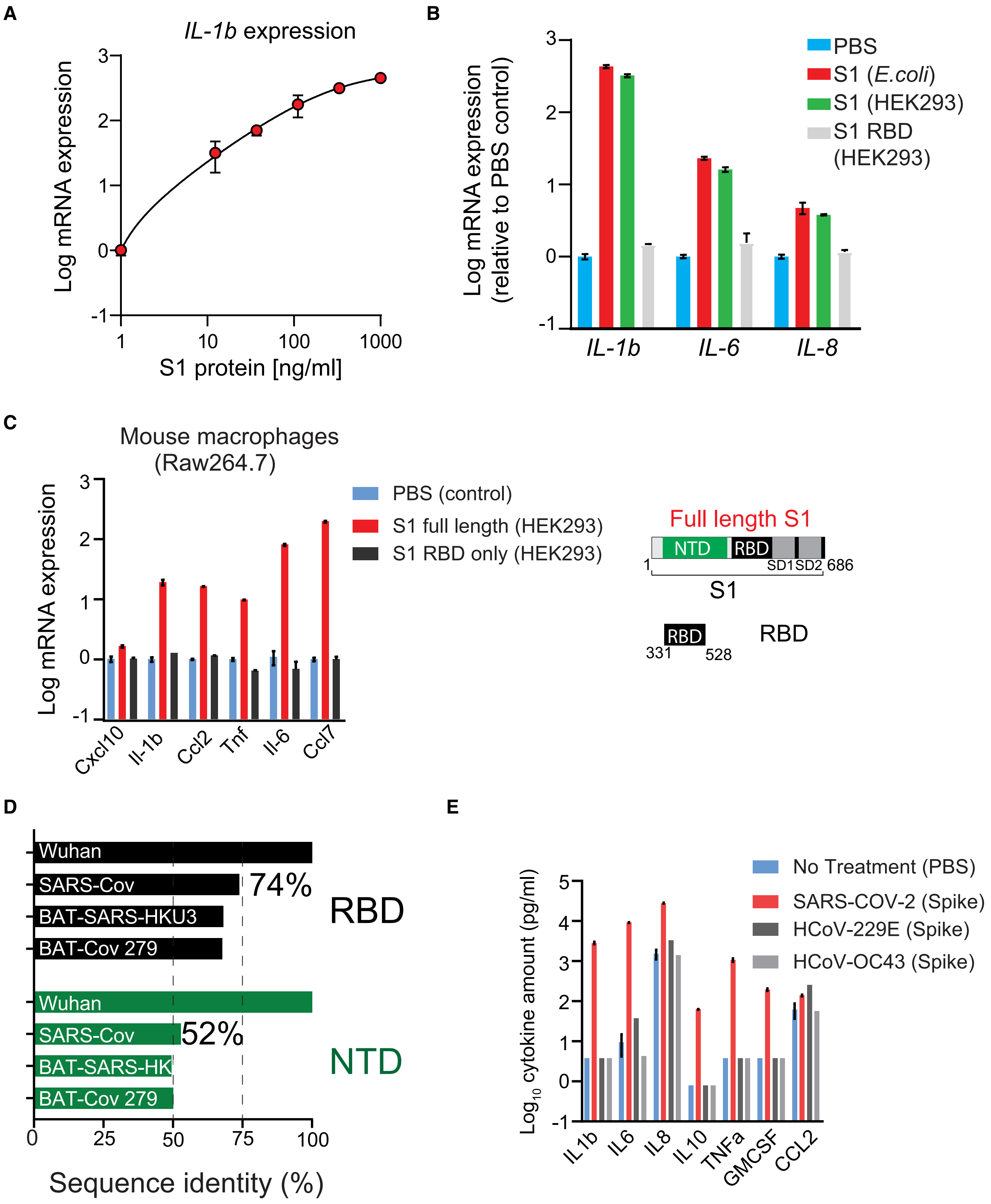
A
IL-1b expression
Log mRNA expression
S1 protein [ng/ml]
1
10
100
1000
B
Log mRNA expression (relative to PBS control)
PBS
S1 (E.coli)
S1 (HEK293)
S1 RBD (HEK293)
IL-1b
IL-6
IL-8
C
Mouse macrophages (Raw264.7)
Log mRNA expression
PBS (control)
S1 full length (HEK293)
S1 RBD only (HEK293)
Cxcl10
Il-1b
Ccl2
Tnf
Il-6
Ccl7
Full length S1
NTD
RBD
SD1
SD2
686
S1
RBD
331
528
RBD
D
Wuhan
SARS-Cov
BAT-SARS-HKU3
BAT-Cov 279
74%
RBD
Wuhan
SARS-Cov
BAT-SARS-HK
BAT-Cov 279
52%
NTD
0
25
50
75
100
Sequence identity (%)
E
Log10 cytokine amount (pg/ml)
No Treatment (PBS)
SARS-COV-2 (Spike)
HCoV-229E (Spike)
HCoV-OC43 (Spike)
IL1b
IL6
IL8
IL10
TNFa
GMCSF
CCL2

Figure EV1.

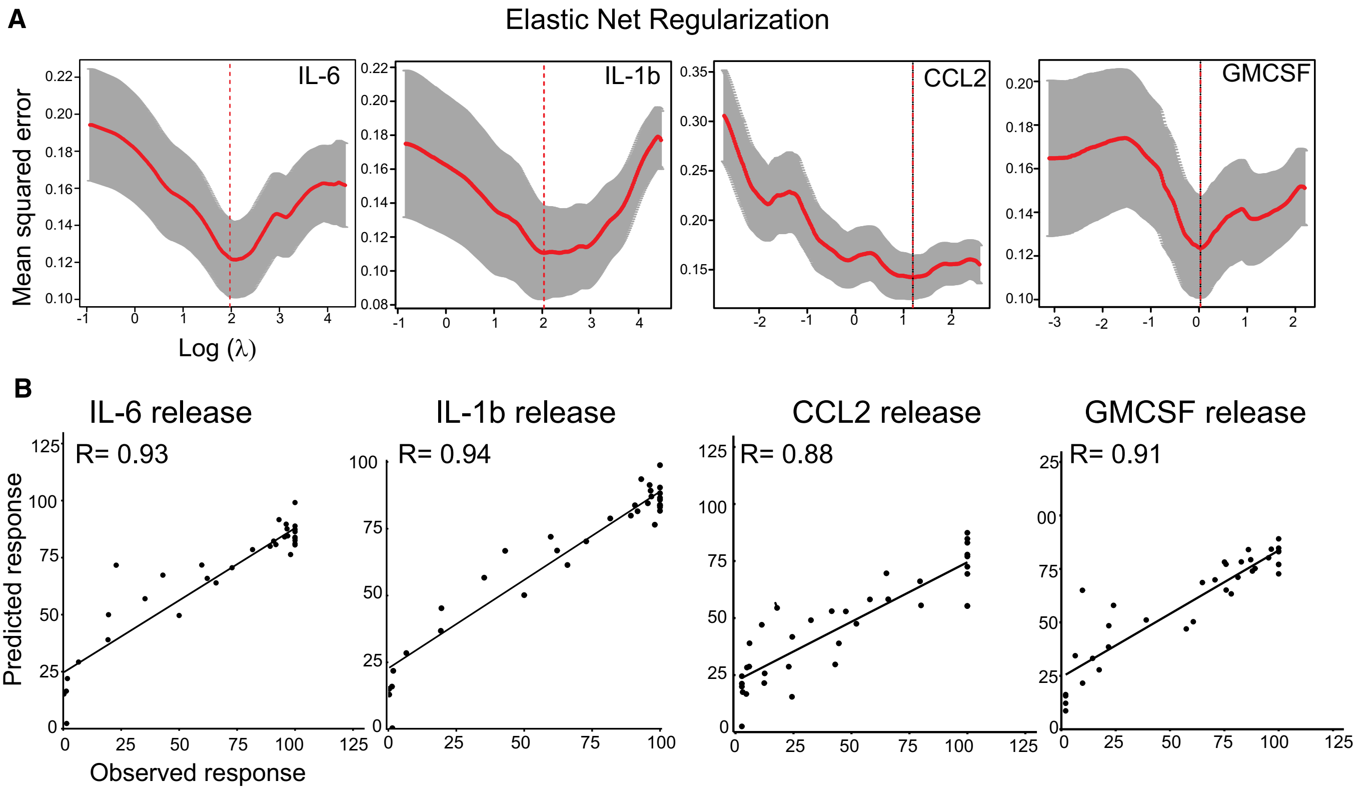

Figure EV2. Applying kinase inhibitor regularization (KiR) to SARS-Cov-2 Spike subunit S1-mediated changes in cytokine release.

A Representative plots showing leave-one-out-cross-validation error using elastic net regularization fit for indicated cytokines. The error bars (gray) represent cross-validation error plus 1 SD. The kinases identified at absolute minima (red dashed line) were termed the most informative kinases.

B Plot showing a correlation between model-predicted and observed response in pooled PBMCs for indicated cytokines.

 ©2021 The Authors

A

Post-Optimization KiDNN Hyperparameters for Each Cytokine Model

Hyperparameters	IL1B	IL6	IL8	IL10	CCL2	GMCSF	TNFalpha
**Activation Function**	ELU	SELU	SELU	SELU	SELU	RELU	ELU
**Batch Size**	4	37	37	32	2	37	1
**Epochs**	350	200	375	100	75	50	125
**Nodes per Hidden Layer**	200	1000	25	25	200	100	100
**Weight Initialization**	Truncated Normal	Variance Scaling	Uniform	Uniform	Uniform	Truncated Normal	normal
**Hidden Layers**	2	2	4	3	5	1	5
**Optimizer**	Adamax	Adam	Adam	Adamax	Adagrad	Adamax	Adagrad

B

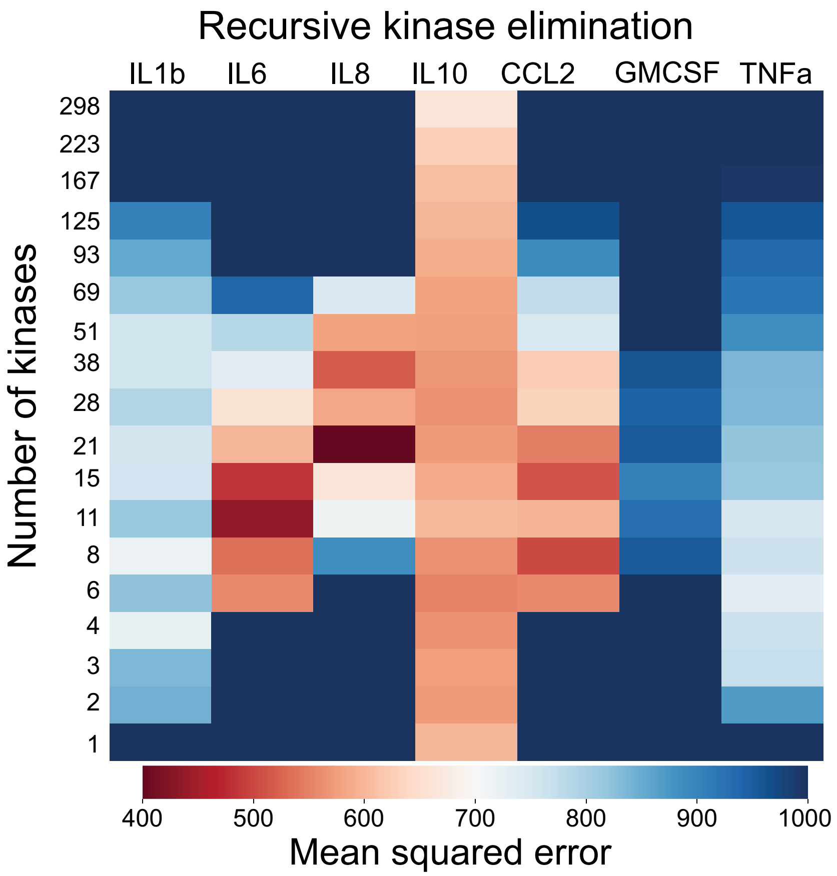

Figure EV3. KiDNN optimization.

A A table showing a set of hyperparameters used for developing KiDNN models for each cytokine. ReLU; rectified linear unit, ELU; exponential linear unit, SeLU; Scaled Exponential Linear Unit, Adagrad; adaptive gradient.

B A heatmap showing mean squared error following the recursive kinase elimination step for each KiDNN model.

© 2021 The Authors

Figure EV4. Model-predicted informative kinases.

A A table showing a list of kinases predicted to be necessary for the release of indicated cytokines by both KiR and KiDNN modeling. Sixteen kinases from this list were chosen for experimental validation (shown in blue).

B Plot showing knockdown efficiency of siRNA targeting indicated kinases compared with scrambled control. Data are presented as means of three technical replicates, and error bars represent SEM.

C Pathway enrichment plot generated using PathwayNet (Park *et al*, 2015) using input from model-predicted kinases.

D Schematic showing known interactions between model-predicted kinases and transcription factors generated using PathwayNet (Park *et al*, 2015).

©2021 The Authors

A

Model predicted 'informative' kinases

CCL2	GMCSF	IL1-b	IL-6	IL-8	TNFa
MAP4K2	STK17	MAP3K8	MAP3K8	MAP3K8	MAP3K8
JAK1	MAP3K8	MAPK14	PTK5	MAP4K2	PTK5
ERBB2	PTK5	EPAH7	MAP4K2	MAP3K3	MAPK14
PTK5	ERK1	ITK	MAP3K3	CDK6	ERK2
CDK5	EPHA7	MEKK2	CDK6	CAMKK2	BRSK2
CLK1	MAP4K2	PRKD1	EPAH7	IRAK1	MAP3K3
PTK2B	TGFBR2	NLK	ERK1	PRKACG	MAP3K10
ACK1	MAP3K3	GSK3B	MAPK12	PTK5	EPHA7
MAP3K8	CDK6	ULK3	LRRK2	PTK2B	LRRK2
EPHA3	MAPK12	VEGFR2	STK17A	CLK1	STK17A

Cytokines

16 Experimentally tested kinases

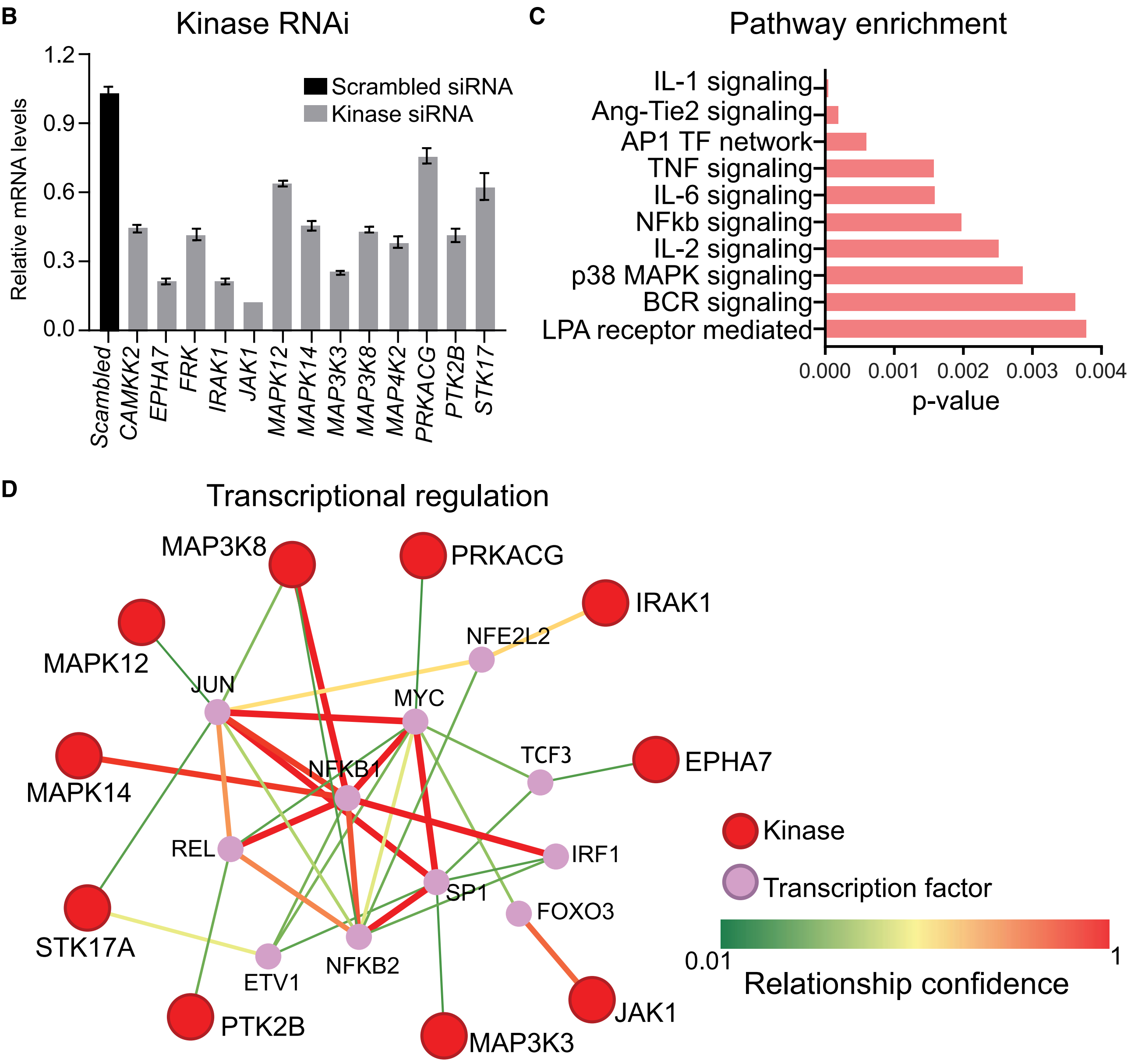

Figure EV4.

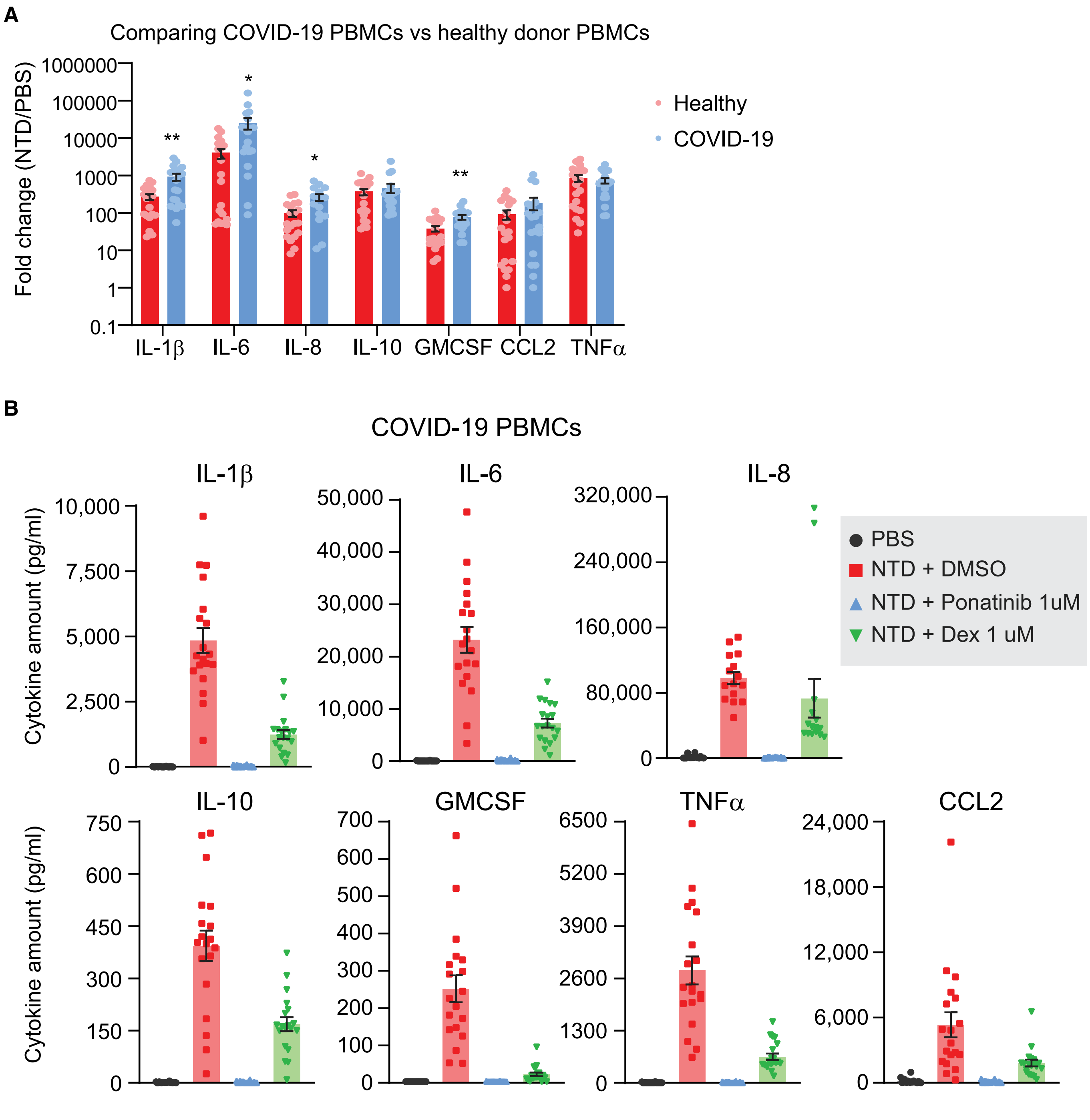

Figure EV5. Ponatinib inhibits NTD-stimulated cytokine release in PBMCs from COVID-19 patients.

A Fold change in the amount of cytokine release in response to NTD stimulation in COVID-19 PBMCs (n = 19) and healthy donor PBMCs (n = 20). Bars represent mean, and error bars denote SEM. *P < 0.05, **P < 0.01, Welch's t-test.

B Plots showing changes in indicated cytokines in response to NTD at 1 μg/ml and ponatinib or dexamethasone in COVID-19 PBMCs for 24 h. Cytokines released in the media were measured by Luminex. Bars represent the mean of 19 individual donors. Error bars represent SEM.

©2021 The Authors

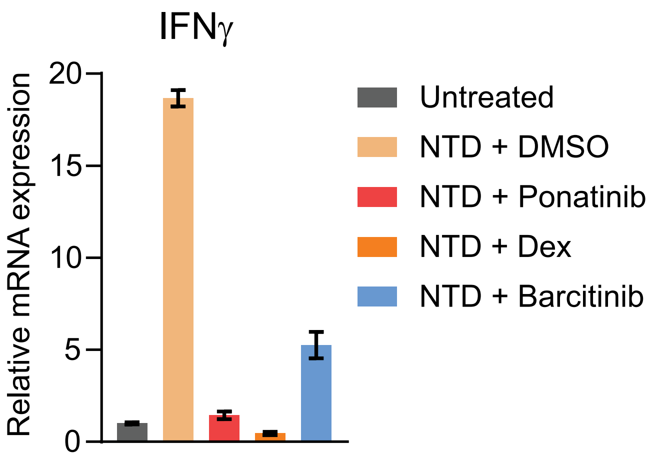

Figure EV6. Ponatinib inhibits NTD-mediated interferon gamma expression in PBMCs.

Changes in the NTD-stimulated expression of *IFNG* in response to ponatinib (125 nM), baricitinib (1,000 nM), or dexamethasone (1,000 nM) in PBMCs. Bars represent the mean of triplicate, and error bars represent SEM.

Figure EV7. Effect of ponatinib on T-cell response.

PBMCs were stimulated with pooled CMV peptides in the presence of indicated drugs in serial dilution from 0 to 250 nM. CMV-specific T-cell response was measured as percentage of AIM^+ cells. Data were background-subtracted against peptide pool diluent (PBS containing 0.01% DMSO) negative control.

A Flow cytometry gating scheme.

B *Left top*, net percentage of AIM^+ ($CD69^+$ $4\text{-}1BB^+$) $CD8^+$ T cells; *Left bottom*, AIM+ $CD8^+$ T cells in the presence of 125 and 250 nM of indicated drugs as a percentage of CMV pooled peptide control. *Right top*, percentage of AIM^+ ($CD69^+$ $CD40L^+$) $CD4^+$ T cells; *Right bottom*, AIM^+ $CD4^+$ T cells in the presence of 125 and 250 nM of indicated drugs as a percentage of CMV pooled peptide positive control. Data are presented as means of three biological repeats, and error bars represent SEM. Dash lines denote 125 nM (a) and 250 nM (b).

C Percentage of viable PBMCs treated with CMV and ponatinib at various concentrations. Data are presented as means of three biological repeats, and error bars represent SEM. Dash lines denote 125 nM (a) and 250 nM (b).

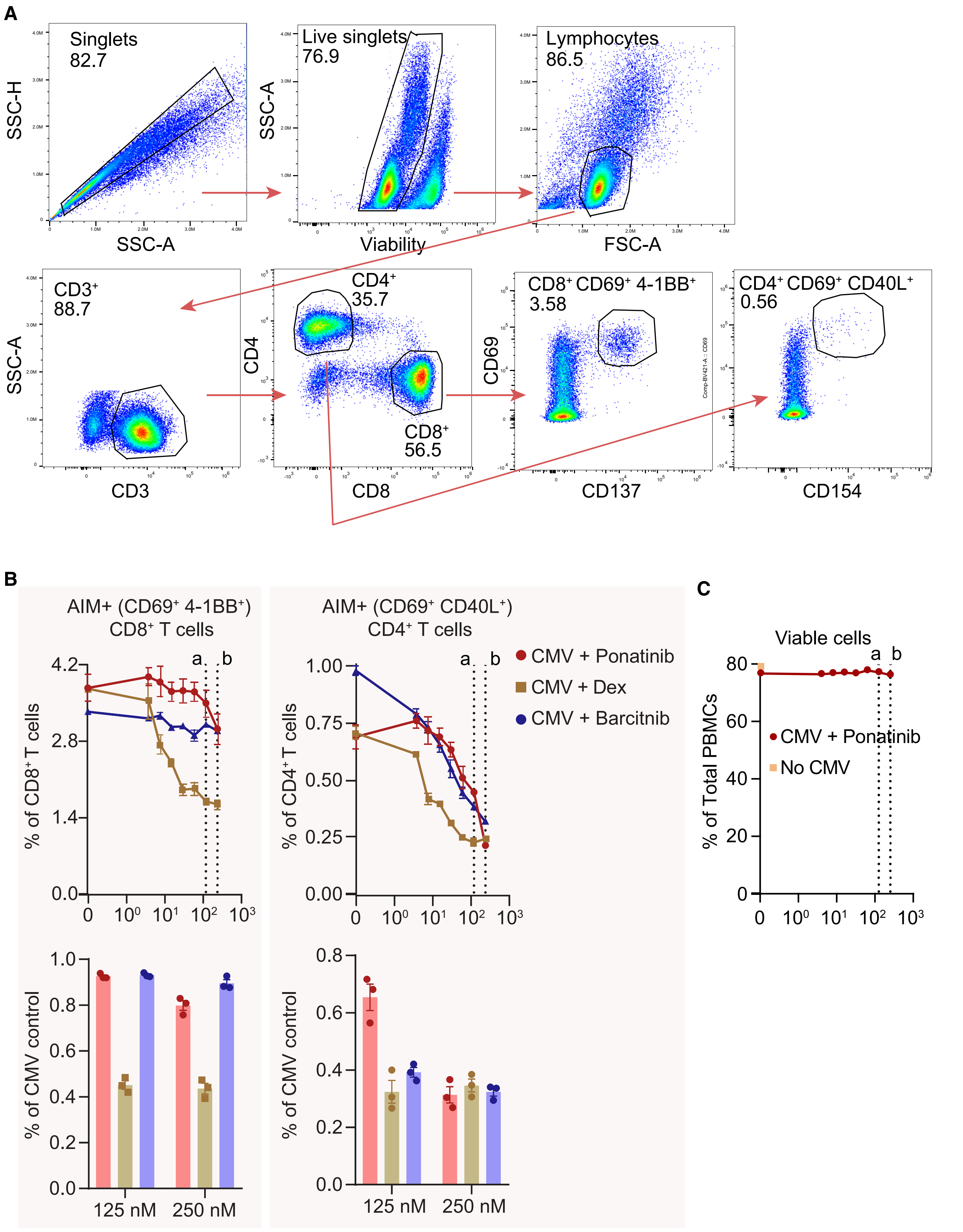

Figure EV7.

 ©2021 The Authors
